# Overlapping of Serotonin Syndrome with Neuroleptic Malignant Syndrome due to Linezolid-Fluoxetine and Olanzapine-Metoclopramide Interactions: A Case Report of Two Serious Adverse Drug Effects Caused by Medication Reconciliation Failure on Hospital Admission

**DOI:** 10.1155/2016/7128909

**Published:** 2016-06-28

**Authors:** Faizan Mazhar, Shahzad Akram, Nafis Haider, Rafeeque Ahmed

**Affiliations:** ^1^Prince Sultan Military College of Health Sciences, King Fahad Military Medical Complex, Dhahran 31932, Saudi Arabia; ^2^King Abdulaziz Medical City, National Guard Health Affairs, Riyadh, Saudi Arabia; ^3^The New York School of Medical and Dental Assistants, Long Island City, NY, USA

## Abstract

Antipsychotic and antidepressant are often used in combination for the treatment of neuropsychiatric disorders. The concomitant use of antipsychotic and/or antidepressant with drugs that may interact can lead to rare, life-threatening conditions such as serotonin syndrome and neuroleptic malignant syndrome. We describe a patient who has a history of taking two offending drugs that interact with drugs given during the course of hospital treatment which leads to the development of serotonin syndrome overlapped with neuroleptic malignant syndrome. The physician should be aware that both NMS and SS can appear as overlapping syndrome especially when patients use a combination of both antidepressants and antipsychotics.

## 1. Introduction

Neuroleptic (NL) or antipsychotic drugs and selective serotonin reuptake inhibitors (SSRIs) are frequently used to treat neuropsychiatric disorders. The introduction of one or both of them when the patient is being treated with another potentially interacting drug sometimes triggers syndromes of varying severity as described in the literature [1–3].

Serotonin syndrome (SS) and neuroleptic malignant syndrome (NMS) are life-threatening adverse reactions caused by serotonergic antidepressants and neuroleptics, respectively. Although NMS is historically associated with classic or “typical” antipsychotics, it is also a potential adverse effect of atypical antipsychotics [4]. The classic clinical features of NMS are hyperthermia, muscle rigidity, cardiovascular instability, elevated levels of creatinine phosphokinase (CPK), tachycardia, tachypnea, diaphoresis, leukocytosis, and altered state of consciousness. Drugs with serotonergic activity, including selective reuptake inhibitors (SSRIs) and monoamine oxidase inhibitors (MAOIs, including linezolid), have a high potential to trigger SS. Clinical manifestations of SS are often mild and consist of confusion, myoclonus, hyperreflexia, and trembling [5]. However, certain forms can be life-threatening. Clinical manifestations of these life-threatening cases may include hyperthermia, muscle rigidity, autonomic dysfunction, shock, status epilepticus, and coma. Its presentation differs from NMS somewhat, which includes the following: (a) neuromuscular symptoms such as tremor and rigidity also occur in the NMS, but features such as chills, ataxia, myoclonus, hyperreflexia, and patellar clonus favor the diagnosis of serotonin syndrome; (b) gastrointestinal dysfunction involves presence of nausea, vomiting, and diarrhea which is a unique feature that is not typical of NMS [6]; (c) changes in mental status and autonomic dysfunction are similar, but the temperature increase is not as high as in the NMS; (d) leukocytosis and rise in serum creatine phosphokinase (CPK) and liver enzymes are inconsistent in NMS and minimally elevated in SS; (e) the course is usually benign and most patients recover between the first and the seventh day after the termination of the causative agent, whereas NMS recovery may take up to two weeks [7]. Distinctive, identical, and overlapping features of NMS, SS, and clinical manifestation sepsis are summarized in Table 1. Thus, SS and NMS have overlapping clinical features. There have been a few cases that reported an overlap between these two syndromes [8, 9].

Similarly to all drug toxicity reactions, SS and NMS are diagnoses of exclusion. Disorders to be ruled out include meningitis, malignant hyperthermia, tumor, viral encephalitis, seizure, acute lethal catatonia, hyperthyroidism, heatstroke, and anticholinergic drug intoxication. Given the similarity in presentation and symptoms of NMS and SS, the most effective approach to distinguish between these two syndromes is to obtain an accurate medication history. These can be avoided by appropriate medication reconciliation to ensure that the prescribed drugs do not interact with drugs that belong to these two groups.

Here we described a rare case of a patient satisfying the criteria of both NMS and SS caused by the failure of medication reconciliation on hospital admission; later the patient was found to have a history of taking two offending agents that interact with drugs given during the course of hospital treatment.

## 2. Case Presentation

A 64-year-old 87 kg man with a past medical history of hypertension for 10 years, type 2 diabetes for 13 years, painful diabetic neuropathy, mild depression, and recent diabetic foot infection (DFI) related hospitalization initially presents to the emergency room with a 2-week history of left lower extremity edema, redness, and pain that limits his normal daily activities. He has a lesion close to his small toe that is macerated and foul-smelling. His physical examination was notable for temperature 38.2°C, blood pressure 145/90 mmHg, heart rate 92 beats/minute, respiratory rate 19 breaths/minute, and 1+ lower extremity edema. His initial laboratory values which were ordered in emergency room include sodium 139 mEq/L, potassium 4.3 mEq/L, BUN 19 mg/dL, SCr 0.9 mg/dL, glucose 180 mg/dL, A1C 8%, WBC 15 × 10^3^ cells/mm^3^, hemoglobin 12 g/dL, hematocrit 36%, and platelet count 290,000 cells/mm^3^.

According to a patient his home drugs include hydrochlorothiazide 25 mg/day, lisinopril 20 mg/day, glyburide 10 mg/day, metformin 1000 mg twice daily, olanzapine/fluoxetine, and aspirin 81 mg/day.

He was admitted to the hospital for the cleansing, irrigation, and surgical debridement of his ulcer. The surgical culture of ulcer specimen was positive for methicillin-resistant* Staphylococcus aureus* (MRSA) with following susceptibilities: Vancomycin (R), Clindamycin (S), linezolid (S), and Trimethoprim/sulfamethoxazole (S).

On the second day of admission he was started on IV linezolid 600 mg every 12 hrs and IV moxifloxacin 400 mg/day for the treatment of DFI. The first dose of antibiotics was uneventful. However, after the second dose of linezolid, he became severely nauseated for which he was given IV metoclopramide at 10 mg IV every 6 hrs.

After 12 hrs patient was constantly nauseated and febrile (38.7°C). He was confused, diaphoretic, and dyspneic. ECG showed tachycardia at pulse 104 bpm without ST or T wave abnormalities; blood pressure was 127/87 mmHg. He started to desaturate gradually (oxygen saturation 84%) and was maintained on oxygen therapy via high flow nasal cannula.

On the third day of the hospital, he continued to desaturate and remained febrile and hypertensive at 160/90 mmHg; blood and urine cultures were ordered and returned negative. An X-ray of the chest was clear. Complete blood count showed leukocytosis.

On neurologic examination, he was rigid asymmetrically in upper and lower extremities and right lower limb showed hyperreflexia with clonus. On neurological examination nuchal rigidity was negative, and both eyes were responsive to light; all signs of meningism were negative. Due to the rigidity of extremities, a blood creatine phosphokinase (CPK) level was checked, and it was 1856 mcg/L. Magnetic resonance imaging of the brain was normal. There was no evidence of epileptiform activity on a 12-hour continuous electroencephalography.

After ruling out all possible infections causes of aforesaid mentioned symptoms and giving the consideration on persistent muscle rigidity, high CPK levels, and causality of patient condition with the metoclopramide administration, a clinical diagnosis of the neuroleptic malignant syndrome was made and metoclopramide was stopped. The patient was treated with IV dantrolene 1 mg/Kg stat dose, carbidopa 25 mg/levodopa 100 mg PO, and baclofen 5 mg PO for rigidity. He was maintained on fluid and IV dimenhydrinate 100 mg q 4 hrs for nausea. Labetalol was used to normalize high blood pressure.

Twelve hours after the dantrolene treatment CPK levels were rechecked; it was 780 mcg/L and physical examination showed improvement in rigidity. However, the patient has still exhibited a high fever with diaphoresis, high blood pressure with labetalol deescalation, consciousness disturbance, and myoclonus. The repeat blood complete count showed leukocytosis. Blood culture was negative. In arterial blood gas analysis, pCO_2_ was 27.2 mmHg and pO_2_ was 69.6 mmHg, sO_2_ 81.3%. He was maintained on oxygen.

Because physicians suspected that, despite the execution of NMS treatment, her persistent increase in blood pressure, hyperthermia, and clonus may have been linked to serotonin syndrome. This information, together with his negative workup, prompts the medical team for medication reconciliation. Thorough medication reconciliation revealed that patient was recently started on olanzapine/fluoxetine by a general practitioner 4 days ago and the last dose was taken by the patient 1 day before arriving at ER.

At this point, after the consultation of a clinical pharmacist, it was decided to discontinue linezolid and maintain the patient on fluid, labetalol 20 mg IV, as needed for the control of blood pressure and cyproheptadine hydrochloride 12 mg PO stat, followed by 4 mg every 6 hours for 24 hours. Clonazepam was given to control myoclonus. After 24 hours all of the patient's symptoms abated except slight fever and mild clonus and hyperreflexia. The patient was discharged from the hospital on the 7th day with a prescription of PO cyproheptadine, PO paracetamol, and PO TMP/SMX with moxifloxacin for DFI treatment.

## 3. Discussion

The case described here followed a complex course. The patient was admitted to the hospital for a care of diabetic foot infection, and he was started on IV antimicrobial therapy which consists of moxifloxacin with linezolid for MRSA. During his course of admission, he was found to have different clinical picture due to either an adverse effect of medication or interactions between different medications. Of note, a careful medication reconciliation in the later stage of hospitalization revealed that the patient was taking SYMBYAX® (3 mg/25 mg), a fixed-dose combination of olanzapine (3 mg), and fluoxetine (25 mg) prescribed by his primary care physician for mild depression. This was an off-label use as SYMBYAX is indicated for treatment-resistant major depression.

Fluoxetine is longest acting SSRIs, and its half-life is up to 7 days. Compared to other SSRIs fluoxetine has more potential to interact with other serotonergic drugs, like linezolid [10–12] in this case. Olanzapine is an atypical antipsychotic drug with low potential for extrapyramidal effects and NMS; however a number of cases of olanzapine-induced NMS have also been reported [13, 14].

In our patient after two doses of linezolid, he presented with severe nausea, hyperthermia, diaphoresis, dyspnea, tachycardia, asymmetric rigidity of limbs, and high blood pressure. Although not as prominent to make a definite diagnosis, these symptoms presented within the onset time of 24 hours after the administration of two serotonergic interacting drugs (fluoxetine owing to its long half-life and IV linezolid administration); this satisfies the Sternbach and Hunter criteria and is therefore consistent with SS at that point. However, given the failure of medication reconciliation and high frequency of gastrointestinal side effects of linezolid, he was treated with the antiemetic metoclopramide for severe nausea. Of note, metoclopramide has a dopamine D2 receptor blocking effect and is a known causative agent of NMS [15, 16].

After four doses of metoclopramide, patients' muscle rigidity worsened as evident by physical examination and plasma CPK levels. Though CPK and temperature were not as elevated as expected to rise in these syndromes, overlap is possible and also reported [9]. Therefore, worsening of these signs is likely due to the interaction between olanzapine and metoclopramide which probably initiate or potentiate the signs of NMS, which started to overlap SS. Despite the discontinuation of metoclopramide and initiation of NMS treatment, patients' symptoms, specifically those of SS (blood pressure, hyperthermia, and clonus), did not remit. This in addition to medication history which enables us to diagnose SS and start its treatment protocol.

In clinical practice, the differential diagnosis between SS and NMS is difficult due to the overlap of symptoms and laboratory data, especially if it is triggered when NL and SSRI are used in combination. Some authors consider that it may be the same underlying process with different clinical manifestations [17, 18]. Nevertheless, the addition of an NL and/or SSRI with interacting drugs or vice versa can trigger severe syndromes, which are clinically very difficult to differentiate. Fortunately, and most importantly their initial treatment is common which consists of stopping the causative agent at an early stage and initiate supportive treatment.

## 4. Conclusion

The review and case presented here is intended to highlight the following: (a) physician being aware that both NMS and SS can appear as overlapping syndrome and consider one of these and even both, overlapping specially when patients use combination of both antidepressants and antipsychotics; (b) being cautious when using these two classes of drugs with other interacting drugs, given our case, as both antipsychotic and antidepressants react with metoclopramide and linezolid, respectively; (c) importance of medication reconciliation on admission; and (d) actively participating in pharmacovigilance.

## Figures and Tables

**Table 1:** Characteristics of neuroleptic malignant syndrome, serotonin syndrome, and sepsis.

		Neuroleptic malignant syndrome	Serotonin syndrome	Sepsis
Precipitated by		Dopamine antagonists	Serotonergic agents	*General* (i) Temperature > 38.3°C or < 36°C (ii) Heart rate > 90 beats/minute (iii) Respiratory rate > 20 beats/minute (iv) Altered mental status (v) Increased fluid balance (>20 mL/kg over 24 hr) (vi) Elevated blood glucose > 140 mg/dL (in absence of diabetes) *Inflammatory* (i) WBC > 12 × 10^3^ cells/mm^3^ or <4 × 10^3^ cells/mm^3^ or >10% immature neutrophils (ii) Elevated plasma C-reactive protein (iii) Elevated plasma procalcitonin *Hemodynamic* (i) Hypotension (SBP < 90 mmHg; MAP < 70 mmHg; or SBP decrease > 40 mmHg in adults or <2 SD below normal for age) *Tissue perfusion* (i) Plasma lactate > 1 mmol/L (ii) Decrease capillary refill or mottling
Onset		Variable, 1–3 days	Variable, <12 hours	
Identical features	Vital signs	Hypertension Tachycardia Tachypnoea Hyperthermia (>40°C)	Hypertension Tachycardia Tachypnoea Hyperthermia (>40°C)	
	Mucosa	Hypersalivation	Hypersalivation	
Overlapping features	Skin	Diaphoresis Pallor	Diaphoresis	
	Mental status	Variable, stupor, coma, alert	Variable, agitation, coma	
	Muscles	“Lead-pipe” rigidity in all muscle groups	Increased tone, especially in lower extremities	
Distinct features	Reflexes	Hyporeflexia	Hyperreflexia Clonus (unless masked by increased muscle tone)	
	Pupils	Normal	Dilated	
	Bowel sounds	Normal or decreased	Hyperactive	

## References

[1] Carbone J. R. (2000). The neuroleptic malignant and serotonin syndromes. *Emergency Medicine Clinics of North America*.

[2] Cassidy E., O'Kearne V. (2000). Neuroleptic malignant syndrome after venlafaxine. *The Lancet*.

[3] Tsai H.-C., Kuo P.-H., Yang P.-C. (2003). Fever, consciousness disturbance, and muscle rigidity in a 68-year-old man with depressive disorder. *Chest*.

[4] Trollor J. N., Chen X., Sachdev P. S. (2009). Neuroleptic malignant syndrome associated with atypical antipsychotic drugs. *CNS Drugs*.

[5] Dunkley E. J. C., Isbister G. K., Sibbritt D., Dawson A. H., Whyte I. M. (2003). The hunter serotonin toxicity criteria: simple and accurate diagnostic decision rules for serotonin toxicity. *Quarterly Journal of Medicine*.

[6] Boyer E. W., Shannon M. (2005). The serotonin syndrome. *The New England Journal of Medicine*.

[7] Dosi R., Ambaliya A., Joshi H., Patell R. (2014). Serotonin syndrome versus neuroleptic malignant syndrome: a challenging clinical quandary. *BMJ Case Reports*.

[8] Demirkiran M., Jankovic J., Dean J. M. (1996). Ecstasy intoxication: an overlap between serotonin syndrome and neuroleptic malignant syndrome. *Clinical Neuropharmacology*.

[9] Nisijima K. (2015). Serotonin syndrome overlapping with neuroleptic malignant syndrome: a case report and approaches for differentially diagnosing the two syndromes. *Asian Journal of Psychiatry*.

[10] Kesavan S., Sobala G. M. (1999). Serotonin syndrome with fluoxetine plus tramadol. *Journal of the Royal Society of Medicine*.

[11] Morales N., Vermette H. (2005). Serotonin syndrome associated with linezolid treatment after discontinuation of fluoxetine. *Psychosomatics*.

[12] Thomas C. R., Rosenberg M., Blythe V., Meyer W. J. (2004). Serotonin syndrome and linezolid. *Journal of the American Academy of Child and Adolescent Psychiatry*.

[13] Ananth J., Parameswaran S., Gunatilake S., Burgoyne K., Sidhom T. (2004). Neuroleptic malignant syndrome and atypical antipsychotic drugs. *Journal of Clinical Psychiatry*.

[14] Filice G. A., McDougall B. C., Ercan-Fang N., Billington C. J. (1998). Neuroleptic malignant syndrome associated with olanzapine. *Annals of Pharmacotherapy*.

[15] Friedman L. S., Weinrauch L. A., D'Elia J. A. (1987). Metoclopramide-induced neuroleptic malignant syndrome. *Archives of Internal Medicine*.

[16] Sternbach H. (1991). The serotonin syndrome. *The American Journal of Psychiatry*.

[17] Bhanushali M. J., Tuite P. J. (2004). The evaluation and management of patients with neuroleptic malignant syndrome. *Neurologic Clinics*.

[18] Kontaxakis V. P., Havaki-Kontaxaki B. J., Christodoulou N. G., Paplos K. G., Christodoulou G. N. (2003). Olanzapine-associated neuroleptic malignant syndrome: is there an overlap with the serotonin syndrome?. *Annals of General Psychiatry*.

